# Aortic Aneurysm with and without Dissection and Concomitant Atherosclerosis—Differences in a Retrospective Study

**DOI:** 10.3390/jcdd11100311

**Published:** 2024-10-08

**Authors:** Andrey V. Suslov, Tatiana V. Kirichenko, Andrey V. Omelchenko, Petr V. Chumachenko, Alexandra Ivanova, Yury Zharikov, Yuliya V. Markina, Alexander M. Markin, Anton Yu. Postnov

**Affiliations:** 1 Petrovsky National Research Center of Surgery, Moscow 119991, Russia; t-gorchakova@mail.ru (T.V.K.); n7610j@rambler.ru (A.I.); yu.v.markina@gmail.com (Y.V.M.); alexander.markin.34@gmail.com (A.M.M.); anton-5@mail.ru (A.Y.P.); 2 Chazov National Medical Research Center of Cardiology, Moscow 121552, Russia; chumach7234@mail.ru; 3 Department of Topographic Anatomy and Operative Surgery n.a. acad. Yu.M. Lopukhin, Pirogov Russian National Research Medical University, Moscow 117997, Russia; 4 Institute of General Pathology and Pathophysiology, Moscow 125315, Russia; omi@bk.ru; 5 Department of Human Anatomy and Histology, I.M. Sechenov First Moscow State Medical University (Sechenov University), Moscow 125009, Russia; dr_zharikov@mail.ru

**Keywords:** thoracic aortic aneurysm, dissection, atherosclerosis, age, gender differences

## Abstract

Background: Thoracic aortic aneurysm is a latent disease with a high risk of death. Today, as data are accumulating, an estimation of the differences in thoracic aneurysm in men and women of different age groups is required. The present study evaluated the type of atherosclerotic aortic lesions in males and females at different ages regarding the presence or absence of aortic dissection. Methods: A retrospective analysis of clinical and morphological data of 43 patients with thoracic aortic aneurysm was carried out. Patients were divided into groups based on the presence or absence of thoracic aneurysm dissection. Results: Our results of a comparative analysis of the age of study participants showed that patients with aneurysm dissection were younger than patients without dissection. In the subgroup of patients with aortic dissection, the mean age was 50.6 years old, and in patients without aortic dissection, the mean age was 55.0 years old. When conducting a frequency analysis using Fisher’s exact test, it was found that in men and women aneurysm dissection was not associated with atherosclerotic lesions of the aorta. Conclusions: In women and men, aneurysm dissection was not associated with stage of atherosclerotic lesions of the aorta regardless of age; no statistically significant differences were found between the groups with and without aneurysm dissection (*p* > 0.05). Dissection of the thoracic aneurysm developed in the absence of severe atherosclerosis of the thoracic aorta. Only 18.6% men and women possessed atherosclerotic plaques of types IV and V.

## 1. Introduction

The aorta is the main vessel that delivers oxygenated blood to all organs of the human body. In postnatal ontogenesis, the aortic wall undergoes a complex and diverse transformation [1,2]. The morphological and functional transformation of the aorta is due to a number of reasons. First, it is associated with the normal growth and differentiation of cells in the aortic wall. Secondly, the transformation of the aorta is the result of a functional adaptation to hemodynamic changes that occur throughout life. Thirdly, transformation of the aorta is the result of the development of pathology of the vessel itself. In practice, there is a simultaneous combination of several causes and factors leading to morphological and functional changes in the aortic wall [3,4,5].

Aortic aneurysm ranks second in the prevalence of aortic diseases after atherosclerosis [6]. The overall incidence of thoracic aneurysm ranges from 5 to 10 people per 100,000 population, with an increasing trend due to improved diagnostic techniques and an increasing aging population [7]. Modern diagnostic and treatment methods have reduced mortality to 21.7% [8].

Aortic dissection is a life-threatening condition, since it leads to the rupture of the aortic wall with the development of heavy bleeding. Approximately 20% of patients with thoracic aortic aneurysm dissection die before the onset of symptoms or diagnosis [9]. The mortality rate in patients with diagnosed thoracic aortic aneurysm dissection is 25% if treatment was not provided within 6 h and 50% if treatment was not provided within 24 h [10,11,12]. A recent study demonstrated sex differences in aortic dissection development [13].

The problem of identifying prognostic markers of aortic dissection in patients with aneurysm remains relevant despite the fact that research in this area has been conducted for quite a long time. Arterial hypertension is considered to be one of the most important risk factors for aneurism development [14]. At the same time, it is assumed that atherosclerosis of the aorta is not associated with the presence of aneurysm and aortic dissection. Atherosclerosis is characterized by damage of the inner layer of the vessel [15,16]. Dissection of the thoracic aortic aneurysm is characterized by progressive destruction of the integrity of the aortic wall, with the formation of a false lumen in the medial layer of the vessel. However, similar mechanisms of vascular smooth muscle cell death were identified both in atherosclerosis and aortic aneurysm and dissection pathogenesis [17]. Therefore, the study of the relationship of the atherosclerotic process of the aorta and thoracic aortic aneurysm is a relevant and important scientific problem in practical healthcare. The aim of this study was to investigate the features of atherosclerotic lesions of the thoracic aorta in patients with thoracic aortic aneurysm, and to reveal the association between the type of atherosclerotic lesion and dissection of thoracic aortic aneurysm in men and women of different age groups.

## 2. Materials and Methods

### 2.1. Study Participants

A retrospective analysis of the clinical and morphological data of 43 patients with thoracic aortic aneurysm was carried out. The study analyzed patients operated on for aneurysm localized in the thoracic aorta: aortic root, ascending aorta, aortic arch, and descending thoracic aorta. Aortic aneurysm wall segments were obtained during thoracic aortic aneurysm repair surgery. The study was conducted in accordance with the Declaration of Helsinki and approved by the local ethics committee of the Petrovsky National Research Center for Surgery (Approval No. 3 of 17 March 2022).

All study participants were operated on electively because of thoracic aorta aneurysm in the aortic surgery clinic of the Petrovsky National Research Center for Surgery from March 2022 to December 2023. Patients who received an emergency operation due to acute aortic dissection were not included in the study. Patients were divided into groups based on the presence or absence of thoracic aneurysm dissection. Aneurysm dissection was determined based on imaging studies, including echocardiography, computed tomography, and magnetic resonance imaging. The criterion for surgery was a diameter of an aneurysm 55 mm or larger; individual factors were considered, and the decision for surgery was made in accordance with current guidelines [18]. Fragments of the wall of the aortic aneurysm were obtained during the operation of aortic aneurysm replacement. Aortic segments removed from patients were immediately fixed in 10% formalin. Then the fixed segments of the aortic aneurysm were processed according to standard techniques and embedded in paraffin. Sections were examined using hematoxylin and eosin staining. Atherosclerotic lesions of the aortic aneurysm wall were assessed based on the classification of atherosclerotic vascular lesions proposed by Stary H.C. 1995 and R Virmani et al., 2000 [19,20]. According to this classification, all atherosclerotic lesions of the aorta were divided into six types, where type I represented the initial changes in the intima of the aorta; type IIa—fatty spots and stripes prone to progression, and type IIb is fatty spots and stripes resistant to progression; type III—intermediate lesion (preatheroma); type IV—atheroma; type Va—fibroatheroma, type Vb—calcified plaque, and type Vc—fibrous plaque; type VI represented complicated lesions of atherosclerotic plaques with ulceration, hemorrhage into atherosclerotic plaques, or thrombosis.

### 2.2. Statistical Analysis

Data processing was performed using the R programming language in R Studio (version 1.2.5042, R Studio, Inc., Richmond Hill, ON, Canada). The Kolmogorov–Smirnov test was used as a statistical test for the normal distribution of values in the Lilliefors modification. To assess the homogeneity of variances of values, the Fligner–Killeen test and the Bartlett test were used. Outliers were determined using the Bonferroni test. Comparative analysis of means was carried out using the Kruskal–Wallis test. For nominal variables, contingency tables were compiled, and the relationship of variables was established using Fisher’s exact test. Correlation between variables was determined using the Spearman coefficient.

## 3. Results

Patients were divided into two groups depending on the presence or absence of thoracic aortic aneurysm dissection. The study included 43 patients, 58% were patients without aneurysm dissection and 42% were patients with aneurysm dissection. The study participants were predominantly men (77%). Among the men, aneurysm dissection was detected in 45% of cases; in the women, aneurysm dissection was detected in 30% of cases. There was no statistically significant difference between the incidence of aneurysm dissection in men (15 cases out of 33) and women (3 cases out of 10) (*p* = 0.385).

The mean age of men in the group without aneurysm dissection was 55.9 (11.9) years, and in the group with dissection 50.0 (8.9) years. In the women, the mean age was 63.0 (11.1) years in patients with aneurysm without dissection, and 44.7 (13.2) years in the group with aneurysm dissection. The data on age distribution in groups of men and women with and without aneurysm dissection are presented in Table 1.

**Figure 1:**
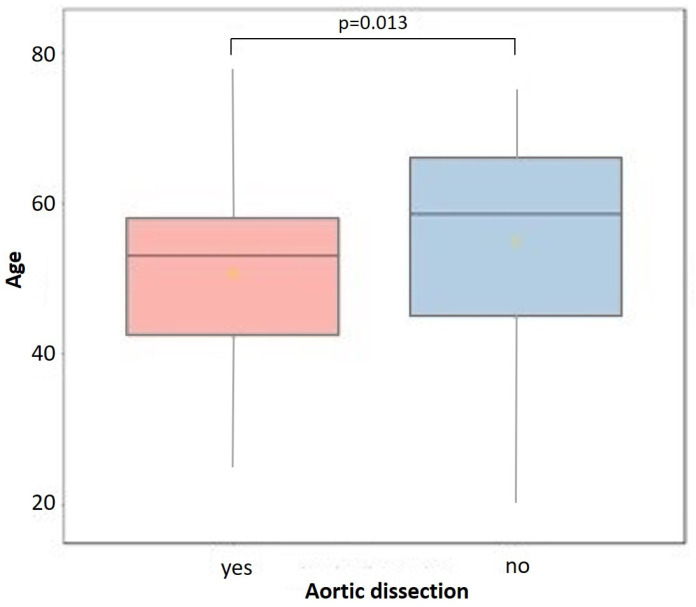
Comparative analysis of patient age for cases with or without aortic dissection according to the Kruskal–Wallis test.

The relationship between nominal variables and the presence or absence of aortic dissection was examined using contingency tables using Fisher’s exact test. Figure 1 presents the results of a comparative analysis of the age of patients in relation to cases of aortic dissection: the mean age was 50.6 (1.7) years old in the subgroup of patients with aortic dissection, and 55.0 (1.7) years old in patients without aortic dissection.

The type of atherosclerotic lesions in men and women was analyzed in groups with and without aneurysm dissection. The data on atherosclerosis types in study participants are presented in Table 2.

**Figure 2:**
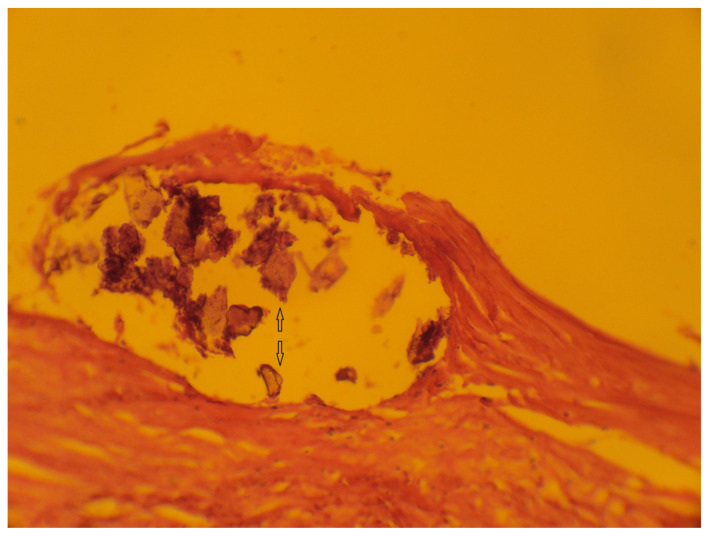
Atherosclerotic plaque (atheroma) of a thoracic aortic aneurysm, type IV. Arrows point to cholesterol crystals in the necrotic core of an atherosclerotic plaque. Hematoxylin and eosin staining ×200.

In the studied samples of men, types I and II of atherosclerotic changes (the early stages of atherosclerosis) were established in 51.2% and 18.1% of cases, respectively. Types IV and V of atherosclerotic changes (the late stages of atherosclerosis) were detected in 18.2% male patients. Type III of atherosclerotic changes was identified in 12.1% of the men. The statistically significant association between the type of atherosclerosis and the presence of aortic aneurysm (*p* = 0.039) was obtained in the group with type II of atherosclerotic changes, which may indicate a relationship between the stage of atherosclerosis and dissection of the thoracic aneurysm. In the other observations, there were no statistically significant differences confirming a possible association between the type of atherosclerotic changes and the presence of thoracic aortic aneurysm dissection. Figure 2 shows a photomicrograph of an atherosclerotic plaque (atheroma) of the aorta in a patient with a thoracic aortic aneurysm without dissection.

It was found that all women with the type I of atherosclerotic changes (40%) did not have aneurysm dissection. Notably, 20% of the women had type II atherosclerotic lesions, and all of them were diagnosed with aneurysm dissection. Dissection of the thoracic aneurysm occurred in half of the cases in women with type III atherosclerosis. There was no aneurysm dissection in women with types IV and V atherosclerosis.

## 4. Discussion

The influence of gender, age, and stage of atherosclerosis on the development of thoracic aneurysm wall dissection was assessed in order to better understand the possible causes of thoracic aneurysm wall dissection. The present study evaluated the type of atherosclerotic aortic lesions in males and females at different ages regarding the presence or absence of aortic dissection. The obtained results confirmed the hypothesis that there is no influence of the atherosclerotic process in the aorta on the development of dissection of the wall of the thoracic aortic aneurysm. In the studied population, aneurysm dissection occurred more often in patients with initial atherosclerotic process, in comparison with patients with severe atherosclerosis (types IV or V of atherosclerotic plaques), which may be explained by the younger age of the study participants with aortic dissection. The difference in the presence of such confounding factors as arterial hypertension, type 2 diabetes, and hereditary aortic syndromes was not statistically significant between the groups with thoracic aorta aneurism and aneurism dissection in men and women, which is why these factors were not considered to be predisposing.

According to recent data, thoracic aneurysm is less common in women than in men, but women with thoracic aneurysm tend to be significantly older [21]. Published data indicate a less favorable prognosis for thoracic aneurysm in women than in men. It has been shown that poor prognosis for aneurysm in women is associated with age [22]. The issue of thoracic aneurysm dissection depending on gender remains controversial. Some studies report a higher risk of thoracic aneurysm dissection in women, while other studies report a higher risk of dissection in men [23,24,25].

The present study was conducted to identify age periods with the highest incidence of thoracic aneurysm dissection. Additionally, a separate analysis of the effect of gender on the incidence of aneurysm dissection in different age groups was performed. It was found that thoracic aortic aneurysm was diagnosed at a later age in women. Thus, the highest prevalence of aneurysm in women was over the age of 60 years, and in men at the age of 45–59 years (average age). The highest incidence of thoracic aneurysm dissection has been established in women under the age of 45 years, and it was equal to 67% compared to other age groups; no women over 60 years of age had aneurysm dissection. In the group of men, the highest percentage of dissection of thoracic aneurysms occurs in the age period of 45–60 years and is 60% relative to other periods.

Studies examining the natural history of atherosclerosis have shown that age is an important factor in the atherosclerotic process [26,27,28]. Our results of a comparative analysis of the age of study participants showed that patients with aneurysm dissection were younger than patients without dissection. In the subgroup of patients with aortic dissection, the mean age was 50.6 (1.7) years old, and in patients without aortic dissection, the mean age was 55.0 (1.7) years old.

To better assess the influence of the atherosclerotic process on the dissection of the aortic aneurysm, the study participants were divided into groups depending on the type of atherosclerotic lesions of the vascular wall.

The study results demonstrated that the majority of patients with thoracic aortic aneurysm had initial atherosclerotic lesions. It was found that the highest frequency of dissection of thoracic aortic aneurysm in men was in patients with types I and II atherosclerotic lesions of the aorta (40% and 34%, respectively). In women, the highest incidence of thoracic aortic aneurysm dissection occurs in type II and III atherosclerotic lesions (67% and 33%, respectively). Note that men and women with types IV and V atherosclerotic plaques accounted for only 18.6% of study participants. The findings are consistent with an earlier study showing a lower prevalence of atherosclerotic lesions in patients with thoracic aortic aneurysm [29].

When conducting a frequency analysis using Fisher’s exact test, it was found that in men and women aneurysm dissection was not associated with atherosclerotic lesions of the aorta; there were no statistically significant differences between male and female groups with and without aneurysm dissection (*p* > 0.05). The only exception was the group of men with type II atherosclerosis; a significant difference was established between the study groups (*p* = 0.039).

### Limitations

This study has limitations. First, it was a retrospective study of patients who underwent surgical treatment. This explains the relatively small sample size and the associated limited amount of data. Second, the etiology of the thoracic aneurysm was not taken into account in the study. The study included hereditary and degenerative forms of the disease. This approach was due to the need to study the atherosclerotic process of the aorta in all patients with thoracic aneurysm. Third, there were no data on the location of the aneurysm and its dissection in specific parts of the aorta (aortic root, ascending section, aortic arch, and descending section). Localization data can show the characteristics of atherosclerosis in various parts of the thoracic aorta. Fourth, the study did not include data from the postoperative period, which was important in clinical practice and the assessment of postoperative mortality. Finally, a limitation of the study was the lack of data on molecular or hemodynamic factors that may underlie the observed associations.

## 5. Conclusions

This study provides new data on the relationship between thoracic aortic aneurysm dissection and the stage of atherosclerosis in men and women at different ages. The results of the study demonstrate a younger age of patients with aneurysm and concomitant dissection and a more frequent presence of type II atherosclerotic lesions than type I in men with aneurysm dissection. The obtained data can serve as the basis for additional diagnostic methods and correction of the timing of elective surgery in women or men in different age groups. Also, the results of the study justify the need to study the features of aneurysm development in the corresponding age groups in order to search for pathogenetic mechanisms of aneurysm transformation leading to dissection.

## Figures and Tables

**Table 1:** Clinical characteristics of study participants.

Characteristics of Participants	Total (n = 43)	Patients without Aneurysm Dissection (n = 25)	Patients with Aneurysm Dissection (n = 18)	Difference, *p*
Gender				
Men, n (%)	33 (77%)	18 (55%)	15 (45%)	
Women, n (%)	10 (23%)	7 (70%)	3 (30%)	
Diameter of aneurism, mm				
Men	58 (10)	58 (10)	58 (9)	>0.05
Women	57 (9)	57 (9)	58 (8)	>0.05
Average age, years				
Men	53 (10.4)	55.9 (11.9)	50.0 (8.9)	0.012
Women	58 (11.8)	63.0 (11.1)	44.7 (13.2)	0.004
Distribution by age groups, men				
up to 45 years, n (%)	9 (27%)	5 (28%)	4 (27%)	>0.05
45–59 years, n (%)	13 (39%)	4 (22%)	9 (60%)	0.025
over 60 years old, n (%)	11 (34%)	9 (50%)	2 (13%)	0.012
Distribution by age groups, women				
up to 45 years, n (%)	2 (20%)	-	2 (67%)	-
45–59 years, n (%)	3 (30%)	2 (29%)	1 (33%)	>0.05
over 60 years old, n (%)	5 (50%)	5 (71%)	-	-
Arterial hypertension				
Men, n (%)	23 (70%)	10 (56%)	13 (87%)	>0.05
Women, n (%)	7 (70%)	4 (57%)	3 (100%)	>0.05
Type 2 diabetes mellitus				
Men, n (%)	13 (39%)	7 (39%)	6 (40%)	>0.05
Women, n (%)	4 (40%)	3 (42%)	1 (33%)	>0.05
Hereditary aortic syndromes				
Men, n (%)	9 (27%)	5 (28%)	4 (27%)	>0.05
Women, n (%)	3 (30%)	2 (28%)	1 (33%)	>0.05

**Table 2:** Stages of atherosclerosis of study participants.

Characteristics of Participants	Total (n = 43)	Patients without Aneurysm Dissection (n = 25)	Patients with Aneurysm Dissection (n = 18)	Difference, *p*
Types of atherosclerosis, total group				
Type I, n (%)	21 (49%)	15 (60%)	6 (33%)	>0.05
Type II, n (%)	8 (19%)	1 (4%)	7 (39%)	0.017 *
Type III, n (%)	6 (14%)	3 (12%)	3 (17%)	>0.05
Type IV, n (%)	2 (4%)	2 (8%)	-	-
Type V, n (%)	6 (14%)	4 (16%)	2 (11%)	>0.05
Types of atherosclerosis, men				
Type I, n (%)	17 (52%)	11 (61%)	6 (40%)	>0.05
Type II, n (%)	6 (18%)	1 (5.5%) *	5 (34%) *	0.039 *
Type III, n (%)	4 (12%)	2 (11%)	2 (13%)	>0.05
Type IV, n (%)	1 (3%)	1 (5.5%)	-	>0.05
Type V, n (%)	5 (15%)	3 (17%)	2 (13%)	>0.05
Types of atherosclerosis, women				
Type I, n (%)	4 (40%)	4 (57%)	-	-
Type II, n (%)	2 (20%)	-	2 (67%)	-
Type III, n (%)	2 (20%)	1 (14%)	1 (33%)	>0.05
Type IV, n (%)	1 (10%)	1 (14%)	-	-
Type V, n (%)	1 (10%)	1 (14%)	-	-

*—significant difference between groups with and without aneurysm dissection, *p* < 0.05.

## Data Availability

Dataset available on request from the authors.
